# Comparing sustainable product hashtags: Insights from a historical twitter dataset

**DOI:** 10.1016/j.dib.2023.109427

**Published:** 2023-07-19

**Authors:** Cristian Toșa, Ari K.M. Tarigan

**Affiliations:** Department of Safety, Economics, and Planning, University of Stavanger, Norway

**Keywords:** Content analysis, Eco-friendly, Consumption perspectives, Climate neutrality, Green products

## Abstract

This data article describes the process of data collection and analysis of Twitter conversations about sustainable products. The dataset contains the IDs of tweets tagged with the hashtags #sustainableproducts, #ecoproducts, #ecofriendlyproducts, and #greenproducts. The time period spans 10 years and includes a total of over 140 thousand tweets from around the world. The article describes the process of obtaining the data using Twarc and the Twitter developer's academic researcher API and describes the preprocessing techniques used to identify keywords, hashtags, topics, and sentiments expressed in the conversations. The analysis identifies key attributes of each sustainable product category as well as commonalities and differences within and across categories. The data have the potential to be reused in future research related to sustainable consumption and production, including further analysis of the sentiments and attitudes expressed in the Twitter conversations and comparison with other social media platforms or survey data. In addition, the data can serve as a basis for marketing strategies and product design by enterprises or organizations seeking to promote sustainable products.

**Specifications Table**

**Table:** 

Subject	Business, management, and decision sciences
Specific subject area	The data article addresses sustainable product consumption patterns in social media with high relevance for researchers, policymakers, and businesses in the areas of entrepreneurship and marketing.
Type of data	Databases (CSV)
How the data were acquired	This data on sustainable products was collected using the Twarc library's command-line tool in Python. In order to search and retrieve the entire archive of historical tweets, the Twitter developer's API account for academic researchers was required and was thus obtained beforehand. Specific Twarc library scripts for data acquisition and conversion were used in Python.
Data format	Raw
Description of data collection	The Twarc command-line tool was used in Python to access historical tweets thorough the Twitter API, using personalized access credentials dedicated for academic researchers. Between July 23 and July 29, 2022, all tweets, excluding retweets, containing the hashtags #sustainableproducts, #ecoproducts, #ecofriendlyproducts, and #greenproducts were collected using keyword-specific queries. There was a total of 141,386 tweets collected and saved as JSON files. For a more convenient data analysis pipeline, they were converted to CSV files using Twarc command-line tool. To comply with Twitter developers’ privacy policy agreement, only the tweet IDs were preserved in raw format and made available for research purposes.
Data source location	The dataset contains tweet IDs corresponding to all English language tweets posted from any country and location. Data and Python code was stored in Open Science Framework (OSF) data repository and was collected and analyzed at the University of Stavanger, city of Stavanger, Norway.
Data accessibility	Repository name Open Science Framework (OSF) Data identification number: 10.17605/OSF.IO/NPW7S Direct link to data: https://doi.org/10.17605/OSF.IO/NPW7S

## Value of the Data

• Our dataset is unique, providing a wider understanding of sustainable products across social media. It contains IDs for more than 140 thousand historical tweets grouped into 4 product categories labeled with the hashtags #sustainableproducts, #ecoproducts, #ecofriendlyproducts, and #greenproducts.
• This dataset has the potential to attract the interest of researchers, enterprises, NGOs, and policymakers. While the characteristics of sustainable products have been studied by researchers in various fields, we anticipate that this dataset will contribute to a deeper understanding of consumption patterns on social media.
• This dataset can be used for sustainable consumption and production related research, including studying characteristics over specific time periods and making comparisons with other social media platforms.

## 1 Objective

The creation of this dataset was motivated by a desire to explore the attributes of sustainable products. Since social media platforms such as Twitter provide a rich source of data on consumer attitudes and behaviors, it is imperative to investigate novel approaches for analyzing and using this data to gain valuable insights into sustainability-related consumer behavior issues. This approach enables a better understanding on the perceptions and discourse surrounding sustainable products among social media users, facilitating the identification of key themes and sentiments associated with these discussions.

This dataset is used to provide insights for marketing strategies and product design aimed at promoting sustainable products. By employing this approach, business entities can gain valuable insights regarding the significance of various characteristics and attributes to consumers, as well as the manner in which these aspects are discussed within the context of social media.

## 2 Data Description

Twitter is a microblogging service that allows people to share updates, news, and information within their network and beyond [1]. Twitter has a rapidly growing user base of over 238 million active users from diverse backgrounds and locations who discuss and disseminate tweets targeted at a broad audience [2]. Twitter API provides unprecedented access to rich data for comprehensive content classification and facilitates businesses' interactions with their customers by enabling users to generate content that better meets customer needs [3]. Twitter users post nearly 500 million tweets daily, making the platform the most popular due to its functionality and allowing users to communicate their thoughts about various products and services [4]. Twitter's academic API allows researchers to conduct scientific research by examining users' tweets [5], making Twitter an ideal platform for this study.

Twarc is a powerful Python-based tool that can help researchers and analysts gain deeper insights into Twitter data [6]. Twarc is a tool that offers flexibility and functionality and provides several utilities. Since Twitter restricts the sharing of Twitter content with third parties [7], Twarc provides the ability to hydrate and dehydrate tweets to reveal and hide sensitive information. Therefore, the stored data in the open access repository OSF consists of dehydrated tweets corresponding to 10,374 IDs for #sustainableproducts, 10,077 IDs for #ecoproducts, 23,787 IDs for #ecofriendlyproducts, and 97,148 IDs for #greenproducts. The tweet IDs are stored in separate text files at the following address: https://osf.io/npw7s. Each of the files stored corresponds to a product category, and each of the files contains the tweet IDs corresponding to that product category. Additionally, Python codes have been uploaded to ease the tasks in data processing pipelines and replicate the methodology in other areas of research. The information in the data files is summarized in Table 1.

**Table 1:** Dataset information on OSF repository.

Storage type	File name	File type	Fields
Raw data	Eco Friendly Products	Text	Tweet ID
	Eco Products	Text	Tweet ID
	Green Products	Text	Tweet ID
	Sustainable Products	Text	Tweet ID
Code files	De_Re_Hydrate_tweets	Python script	-
	Search_download_tweets	Python script	-
	Preprocess_tweets_pipeline	Python script	-
	Tweets_analysis_model	Python script	-

**Fig. 1:**
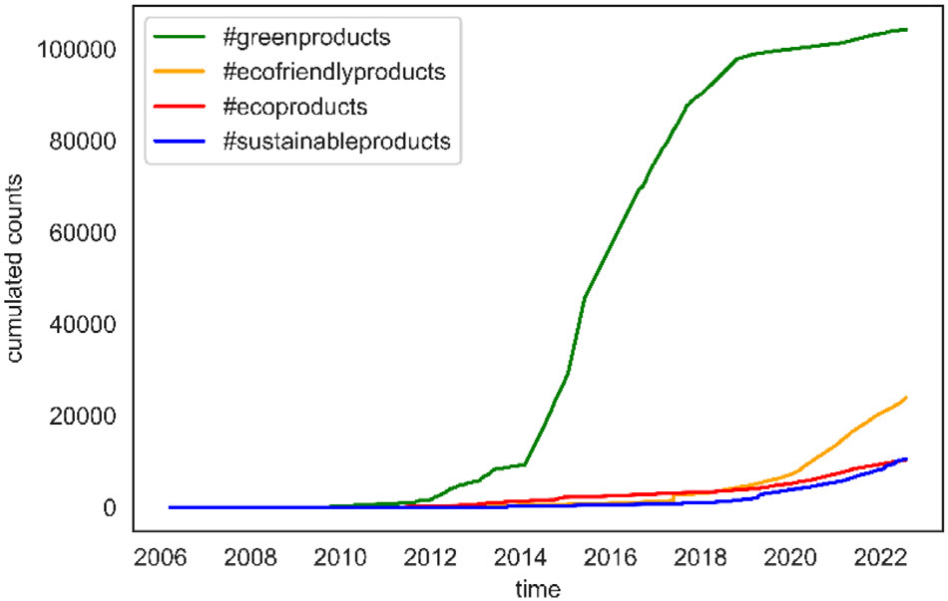
Cumulated counts for tweet categories.

Datasets were collected via the Twitter Academic API using the open-source Python library Twarc [6], a command-line tool for collecting tweets. Data search and collection were initiated by authorizing Twarc to search for and retrieve Twitter data. The search and data collection were conducted between July 23 and July 29, 2022. We used the Twarc command-line tool to perform a search for the available number of tweets. The search criteria yielded a total of 141,386 tweets that were subsequently collected: 10,374 for #sustainableproducts, 10,077 for #ecoproducts, 23,787 for #ecofriendlyproducts, and 97,148 for #greenproducts. Any tweet that contains one of those hashtags was downloaded, and the cumulated number of historical tweets for each group is presented in Fig. 1. We need to mention that our study on sustainable products emphasizes the acquisition of archived historical tweets instead of analyzing real-time data. The deliberate choice enabled us to examine a particular temporal period and document the characteristics pertaining to sustainable products within that timeframe.

Table 2 reveals the characteristics of each of the first conversations tweeted for each of the product categories. Some of the tweet text was removed or corrected to eliminate any reference to user data or websites, while the essential parts of the messages were preserved and presented. The first tweet among the considered product categories is related to #ecoproducts and was created on November 3rd, 2008.

**Table 2:** First tweets on sustainable product categories.

Product category	Part of tweet text	Date tweeted (YYYY-MM-DD)
#sustainableproducts	“… *We encourage you to shop our #sustainableproducts* …”	2009-10-05
#ecoproducts	“*It is a bad feeling to find out that our cup supplier (#ecoproducts) is out of cups!* …”	2008-11-03
#ecofriendlyproducts	“*World First Environment-Friendly Robot Unveiled* … *#ecofriendlyproducts*”	2009-11-26
#greenproducts	“*Anyone know of software that groups do[c]uments and email electronically instead of using paper based files?* … *#greenproducts*”	2009-02-25

## 3 Experimental Design, Materials and Methods

**Fig. 2:**
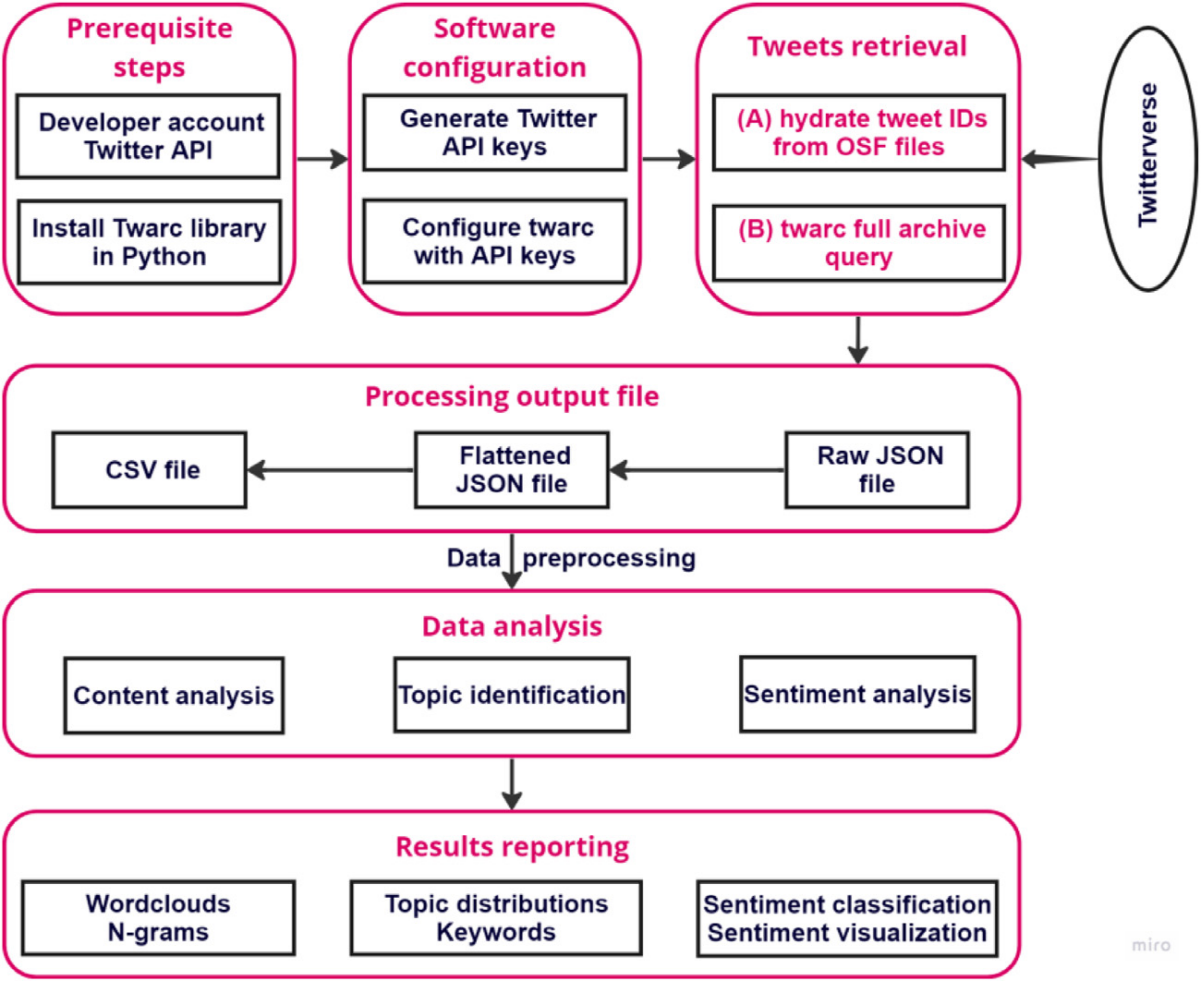
Methodological approach.

The replication of this research can be done twofold: (A) replicate the methodology by retrieval of OSF repository data [8], hydrate the raw data to obtain the complete CSV files on tweets [6], or (B) use Twarc [6] and Twitter API to download other tweets of interest to the researcher. Then, proceed with the data analysis and results reporting according to Fig. 2.

### 3.1 Raw data collection

#### 3.1.1 Method A

In accordance with the terms of use and privacy policy of Twitter developers, the sustainable products dataset only contains tweet IDs. As a prerequisite, a Twitter API developer account is needed to obtain the Twitter API credentials [7]. A configuration of Twarc is necessary using Twitter API credentials. After downloading the raw data files from the OSF repository [8], these can be hydrated locally using Twarc [6] in order to obtain the original tweets with other metadata. After the file containing the tweet IDs are processed in Python with Twarc library, the returned tweets and their metadata are by default in json format. However, it may also be configured to return data in other formats, such as csv.

After hydration, each of the resulting data frames has headers, allowing for the necessary steps to engage in data processing.

#### 3.1.2 Method B

As stated previously, a Twitter API developer account is needed to obtain the Twitter API credentials [7], followed by Twarc configuration in Python. Setting up the search parameters entails identifying the specific requirements that will be utilized to select and retrieve the desired tweets. As stated in Twarc user manual [6], the researcher is instructed to establish search parameters, including hashtags, keywords, usernames, geolocation, or other filters. The retrieval of tweets will be initiated by employing the functionality of Twarc, which will be used to retrieve tweets based on the specified search parameters. The downloaded tweets are by default in json format, but Twarc can convert the raw files and return the data in csv format as well.

### 3.2 Data pre-processing

Preprocessing of Twitter data retrieved with either method (A) or (B) is an important step to solve the problem of noise in the data [9] and can improve the accuracy of text processing operations [10]. Pre-processing included restricting tweets to English and removing punctuation, stop words, hyperlinks, and uninterpretable characters [11]. We then performed the tokenization, stemming, and lemmatization of words [12]. Spaces were removed from the cleaned text, and duplicates in the tweet's text were removed from the database. After preprocessing and deduplication, we obtained the final sample of tweets, shown in Table 3.

**Table 3:** Results of tweet preprocessing.

Group	Total number of tweets	Tweets after preprocessing and cleaning	Percentage of the final dataset (% of total)
#sustainableproducts	10,374	5,001	48.21%
#ecoproducts	10,077	5,394	53.53%
#ecofriendlyproducts	23,787	14,980	62.98%
#greenproducts	97,148	8,432	8.68%

### 3.3 Data analysis

In this section, several steps are presented, as revealed in Fig. 2. First, a descriptive analysis was performed that included users and tweet text analysis. The user analysis showed the most active and visible users on Twitter. Second, content analysis included term frequency, hashtag analysis, topic analysis, and sentiment analysis. Each of these steps will be described below.

#### 3.3.1 User analysis

User analysis shows that 2,976 authors delivered a total of 5,001 #sustainableproduct tweets, 2,026 authors delivered 5,394 #ecoproduct tweets, 4,878 authors delivered 14,980 #ecofriendlyproduct tweets, and 3,620 authors delivered 8,432 #greenproduct tweets.

#### 3.3.2 Term frequency and hashtags

**Fig. 3:**
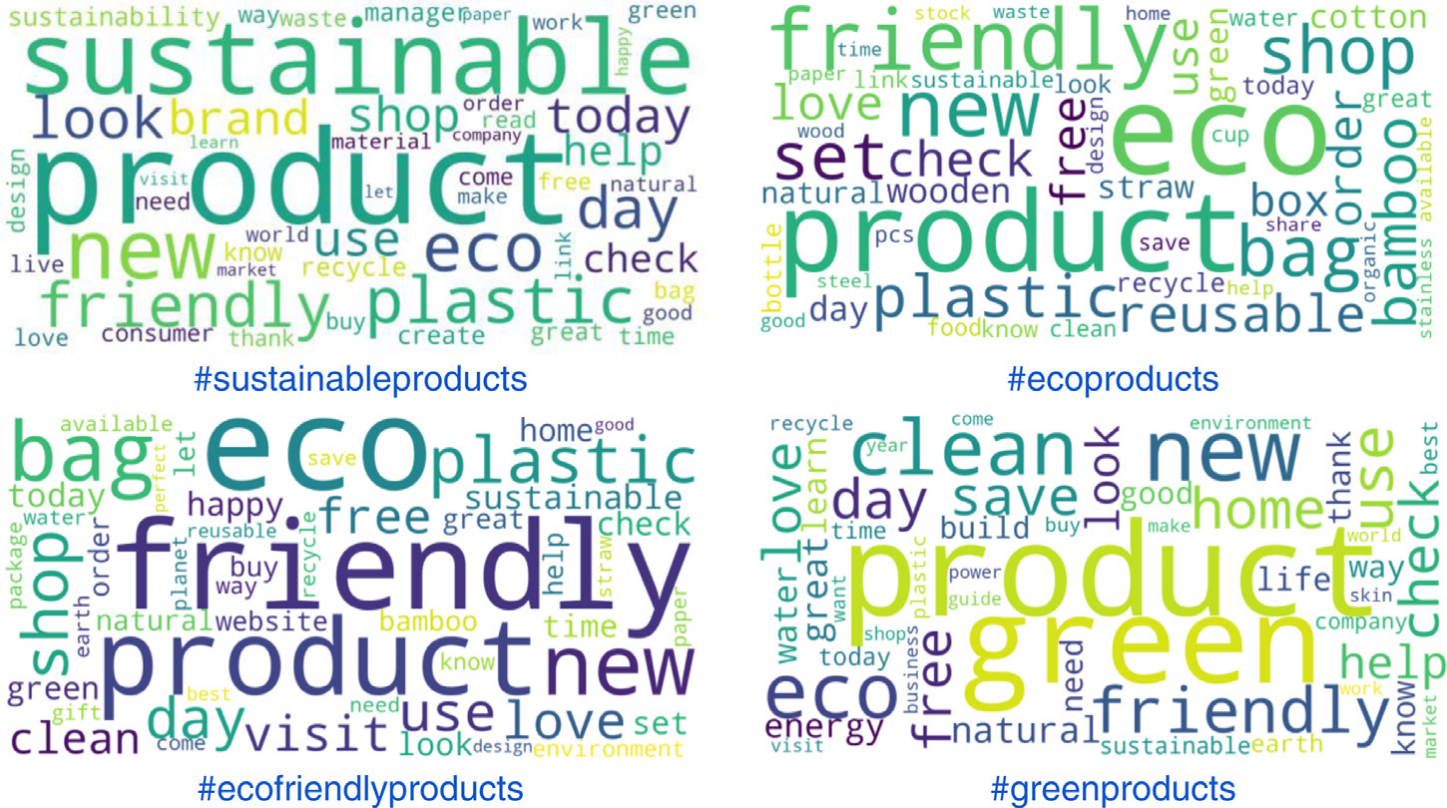
Word clouds for tweet categories.

The use of content analysis, typically used for analyzing text documents, is a valid, rigorous, reliable, and replicable research method [13]. In this study, we created word clouds using the text body of tweets related to four different product categories. Fig. 3 depicts the word clouds for tweets with the hashtags #sustainableproducts, #ecoproducts, #ecofriendlyproducts, and #greenproducts.

In addition to the term frequency analysis, we extracted all the hashtags that appeared in tweets and analyzed them in Python. Some of the most frequently occurring hashtags are presented in Table 4, along with their frequency.

**Table 4:** Hashtag analysis.

#sustainableproducts	#ecoproducts	#ecofriendlyproducts	#greenproducts
sustainableproducts (4,458)	ecoproducts (4,668)	ecofriendlyproducts (14,023)	greenproducts (6,331)
sustainability (654)	ecofriendly (1,221)	ecofriendly (4,805)	greenproduct (2,144)
ecofriendly (613)	ecoproduct (753)	ecofriendlyliving (1,356)	ecofriendly (865)
sustainableproduct (550)	design (608)	gogreen (1,338)	green (533)
sustainable (456)	sustainable (402)	ecofriendlyproduct (1,328)	greenliving (524)
sustainableliving (307)	sustainability (297)	sustainability (1,230)	sustainability (519)
renewableenergy (254)	plasticfree (286)	sustainable (1,149)	environment (295)
cleantech (228)	organic (266)	sustainableliving (1,019)	saveourplanet (269)
ecofriendlyproducts (226)	zerowaste (252)	zerowaste (878)	sustainable (264)
productmanagement (226)	ecotherapy (244)	environment (689)	gogreen (227)

#### 3.3.3 Topic identification

We utilized TweetNLP [14], an integrated package for natural language processing (NLP) platforms for social media. This approach involves the association of a given input text with a specific topic from a predefined set of categories based on Twitter trends [14]. According to the analysis, most tweets were assigned to the topic of business and entrepreneurs, with 31.85% share within #sustainableproducts tweets, 20.97% within #ecoproducts tweets, and 24.47% within the #greenproducts tweets. For #ecofriendlyproducts, the share of topics within business and entrepreneurs is 18.16%, while the highest share is represented by the diaries and daily life topic, with a share of 22.66% out of the total tweets.

#### 3.3.4 Sentiment analysis

We used RoBERTa, a pre-trained language model, to perform sentiment analysis for the tweets in our study [15], and Textblob [16], due to its overall good performance when compared to other sentiment classification tools [17]. RoBERTa is based on the Bidirectional Encoder Representations from Transformers (BERT) method [18], and it has been improved several times to increase its accuracy in determining sentiment in tweets [19]. By analyzing the output of the model, we assigned a positive, neutral, or negative label to each tweet to describe its sentiment. Table 5 depicts the distribution of tweets for each product category across positive, negative, and neutral sentiments, and shows the difference between RoBERTa and Textblob. Notably, when compared to the other categories, #ecoproducts tweets had the largest number of positive tweets (48%) when assessed with RoBERTa. On the other hand, the #sustainableproducts, #ecofriendlyproducts, and #greenproducts tweets had around 60% neutral sentiment. Of all product categories, the #greenproducts category received the greatest share of negative-sentiment tweets (4.36%) and the lowest share of positive sentiment tweets (29.49%).

**Table 5:** Sentiment analysis with RoBERTa and Textblob.

	#sustainableproducts (5001 tweets)		#ecoproducts (5394 tweets)		#ecofriendlyproducts (14980 tweets)		#greenproducts (8432 tweets)	
Sentiment	RoBERTa	Textblob	RoBERTa	Textblob	RoBERTa	Textblob	RoBERTa	Textblob
Positive tweets (%)	1736 (34.71)	2294 (45.87)	1693 (31.38)	2562 (47.50)	5566 (37.16)	7856 (52.45)	2486 (29.49)	3512 (41.65)
Neutral tweet (%)	3114 (62.27)	2019 (40.37)	3548 (65.78)	2246 (41.64)	8984 (59.97)	5479 (36.57)	5578 (66.15)	3475 (41.21)
Negative tweet (%)	151 (3.02)	688 (13.76)	153 (2.84)	586 (10.86)	430 (2.87)	1645 (10.98)	368 (4.36)	1445 (17.14)

Textblob results exhibit different results, tending to balance the shares among the positive and neutral sentiment categories and increasing the shares of the negative ones. Conversations labeled with #sustainableproducts and #ecoproducts exhibit similar shares of positive tweets, while #ecofriendlyproducts have the highest share of positive tweets (52.45%). Similar to sentiment assessment done with RoBERTa, #greenproducts tweets assessed with Textblob received the greatest share of negative-sentiment tweets (17.14%) and the lowest share of positive-sentiment tweets (41.65%).

## 4 Prospective Use of Methodology and Data

The current methodology described in this study was applied to four tweet datasets on #sustainableproducts, #ecoproducts, #ecofriendlyproducts, and #greenproducts, providing aggregated insights on content, topics, and sentiments. We anticipate the datasets hold potential for further analyses and can be expanded to explore both current and additional datasets. For example, it would be interesting to examine variations in tweet characteristics across distinct time periods and compare them across different product categories. This methodology stands for further endeavors to study sustainable production and consumption patterns on social media.

## 5 Important Consideration

The Twitter API requires being used in order to obtain data according to the research article's strategy. However, as of March 27, 2023, Twitter deprecated the tool we employed [7], making it difficult for other academics to apply our findings without subscribing to the premium Enterprise tier [20]. We are conscious that doing so restricts access to the data and makes it more challenging for academics to use open access resources to replicate our findings. To be consistent with the values of open science and to encourage greater engagement in academic research, we do, however, remain optimistic that Twitter will eventually reinstate the prospect of free data access through Academic Research access.

## Ethics Statements

This study was conducted in accordance with ethical guidelines and regulations. This study involved the analysis of publicly available Twitter data. No informed consent was required because all the tweets analyzed were already publicly available. We ensured that participant data was fully anonymized and that no personal information was included in the dataset. We also adhered to the Twitter platform's data sharing policies and terms of use. We obtained permission from Twitter to use the Twitter programming interface for academic research.

## CRediT authorship contribution statement

**Cristian Toșa:** Conceptualization, Investigation, Methodology, Writing – original draft, Writing – review & editing. **Ari K.M. Tarigan:** Resources, Writing – review & editing, Supervision, Project administration.

## Data Availability

Twitter data on sustainable products (Original data) (OSF).
